# Investigation of Relationship Between Spatial Distribution of Medical Equipment and Preventable Mortality

**DOI:** 10.3390/ijerph16162913

**Published:** 2019-08-14

**Authors:** Beata Gavurova, David Tucek, Viliam Kovac

**Affiliations:** 1 Technical University of Košice, 04001 Košice, Slovak Republic; 2 Research and Innovation Centre Bioinformatics, University Science Park Technicom, 042 00 Košice, Slovak Republic; 3 Faculty of Management and Economics, Tomas Bata University, 76001 Zlín, Czech Republic

**Keywords:** medical equipment, computed tomograph, magnetic resonance imaging scanner, standardised mortality rate, preventable mortality, regional disparity, health policy, cluster analysis, dendrogram, heat map

## Abstract

The aim of the study is to investigate the relationship between the spatial distribution of the selected medical equipment and the preventable mortality rate in the regions of the Slovak Republic. The main analytical approach is carried out through the cluster analysis based on a Euclidean distance technique in order to get similarity of the administrative divisions in form of a district and a pseudot2 approach aimed at the determination of a number of the districts in a cluster. A number of medical equipment had a rising tendency from the year 2008. The most extreme position according to a localisation distribution of the computed tomographs and the magnetic resonance imaging scanners is held by the Košice IV District at the level of 7.50630. From an angle of view of the preventable mortality, the Piešťany District holds the most extreme position peaking at the level of 10.97969 for the female sex and the Kežmarok District with the value of 9.44088. The study has the significant dissemination outputs for health policy interventions, especially to draw up regional health plans for computed tomography and magnetic resonance imaging deployment, mainly in locations with a high preventable mortality rate for both sexes.

## 1. Introduction

In the last decade, attention in measuring and evaluating the healthcare system has increased significantly in both the professional sphere and the scientific sphere. This topic is the subject of many research studies, being studied at a national level as well as a regional level in order to capture regional disparities and uncover critical bottlenecks in the healthcare system. Measurement of the efficiency of the healthcare system is methodologically very complex, due to variations in individual systems, different infrastructure, market dynamics, epidemiology, and demography of the country. Demographic forecasts are not optimistic and indicate an increasing prevalence of demand for healthcare as well as its cost. The aging of the population, the introduction of new diagnostic and treatment technologies gradually increase health spending of the countries. Strict budget constraints put pressure on policymakers and other stakeholders to introduce more systematic and efficient ways of measuring and evaluating healthcare systems in order to improve the health of the population and responsibility, management and resource efficiency in the healthcare system of the country. Quantification of the healthcare system efficiency is a cardinal problem in a majority of the countries. As several research studies have declared, the Slovak Republic healthcare system offers a wide scope of opportunities for improvement with the intention of enhancing efficiency [1,2,3,4].

In recent time, the Ministry of Health of the Slovak Republic has introduced the concept of stratification of hospitals, whose aim is to significantly improve institutional healthcare by the year 2030. So far, there are major differences in the quality of healthcare provided by the healthcare facilities in the Slovak Republic. This concerns the mortality rate, reoperation, and rehospitalisation of patients. Exploring the causal relationships between these components and the factors that affect them is also at the forefront. Finding more effective and systematic ways of measuring and evaluating healthcare systems requires discovering a number of analytical trajectories that relate not only to outcomes such as mortality rate, reoperation, and rehospitalisation, but also to the process parameters. This means the investigation of the effects of inputs—in this case, the deployment of selected medical equipment—on outcomes that are represented by preventable mortality in the studied area. This research trajectory makes it possible to evaluate the optimisation of healthcare availability in relation to its demand structures in the individual regions in the terms of healthcare requirements for different age categories, specialised and long-term healthcare, and the other specific areas. These fundamental facts create the motivation to carry out this study, whose main objective is to explore the relationships between the spatial distribution of medical equipment and preventable mortality. This type of mortality is selected because of an orientation in computed tomography and magnetic resonance imaging, which play an important role in a diagnostic process.

The international importance of such research lies primarily in comparative aspects. There are clear trends in the healthcare systems, especially health and economic aspects of the usage of the medical equipment, while its localisation at a regional level as well as a national scale is subject to centralisation or decentralisation within the national healthcare system. The health needs of the regions must reflect the demographic characteristics of the population, the prognosis of its development, the economic and social aspects affecting health behaviour and thus, the demand for healthcare too. It is also necessary to examine the availability of healthcare not only in the terms of geographic and economic parameters, but also regarding the distribution of the specialised medical equipment, whose nonoptimal distribution may cause fluctuations in the development of mortality of the various diseases.

The medical equipment is one of the determinants of the healthcare quality and it also influences the accessibility of the healthcare provision. Usually, the factors like numbers of patients, doctors, beds, and so forth, in the context of the development of the standardised mortality rate are often analysed, but there is a lack of the studies aimed at the preventable mortality with relation to an investigation of their relationship. Such research is done with an assumption that healthcare service is provided at the highest level as it could be at the time of the analysis. Hence, this analysis is aimed just right on the medical equipment and especially, the two most used pieces of the medical equipment aimed at diagnostic purpose. The essential aim of the analysis is to provide a valuable platform for national and regional policymaking to develop strategic framework concepts as well as to establish accurate criteria to identify the necessary primary and secondary prevention programmes for the region and to regulate the availability of healthcare that has a significant impact on preventable mortality.

The structure of the paper is common as, firstly, it offers a general entrance into the topic in a form of the fundamental introduction. Secondly, the Background section involves several scientific sources that create a comprehensive literature review of the issues related to the examined topic. Thirdly, the Data and Methodology section offers information about the data inputted into the analysis and also about the methods and the approaches applied in order to get a precise desired outcome determined at the end of the Introduction section. Fourthly, the Results section introduces the analysis outcome followed by the fifth section, Discussion, where the main results are discussed with the other studies in this field. Finally, the Conclusion section offers substantial findings and, hence, it summarises the whole analysis and introduces potential further research.

## 2. Background

A number of strategies aimed at achieving an optimum combination of computed tomograph investments in the South Side of Chicago are explored in the study applying a linear programming technique [5]. The results of their analysis show that the proper distribution of computed tomographs is equally important for cost-cutting processes as well as ensuring the necessary numbers. The overcapacity of any of the computed tomographs does not mean the inefficiency of the health care processes. Research findings point to the importance of monitoring the economic processes of healthcare as a system to ensure that it is both accessible and efficient.

An increase in the number of computed tomographs during the explored period led to a rise of the regional differences in their deployment and usage is conclude in another study [6]. To increase the efficiency of their use, the authors propose to create a regional computed tomograph system for their optimum regional distribution and though, their efficient usage. According to the economic calculations, an increase in the number of computed tomographs may result in lower overall costs within the regional computed tomography system.

The experience of the health plan operator with the accreditation of imaging services and the demand for a supply of new services is offered in the study [7] with an application of the so-called Roemer’s Law [8]. They apply the data from the period beginning in the year 1998 and ending in the year 2004, which they use computed tomographs and magnetic resonance imaging scanners as a part of the health system plans during. The authors express these pieces of the medical equipment have the potential to generate increased demand for health services in their analysis outcome. However, the innovations in the health processes can boost their usage without increasing an actual demand for health services.

Also, the impact of a geographic approach on the late breast cancer diagnosis is investigated in the study [9]. In the period from the year 2004 to the year 2006, research was conducted on 164,619 women aged 40 years and over in the ten states of the United States of America. Although a geographical approach to mammography is not a significant predictor of the breast cancer diagnosis at a later stage, women in very poor areas, or without health insurance are at the greatest risk of diagnosing breast cancer at a later stage regardless of geographical location and they cannot benefit from targeted health interventions.

The usage of imaging techniques in stroke suspected in Scotland is investigated in another study [10]. The subject of their interest is a geographical approach aimed at the analysis of geographical and digital demographic data through the geographic information systems. They study access to imaging techniques for patients with stroke during working hours. The authors report that radiological departments are fully exploited, but during weekends in many regions, the availability of computed tomography services is limited by vascular stroke cases. It is also an appeal to policymakers to reflect on these findings, and to deal more widely with the availability of computed tomography examinations even on weekends.

There are the authors who investigate geographic aspects in the distribution of medical equipment from a microeconomic point of view and the other authors from a macroeconomic point of view. There are several interesting findings offered by the study taking into consideration a macroeconomic point of view [11]. The authors examined the efficiency of a decentralised approach to healthcare in South Africa in the period from the year 2013 to the year 2016. According to their findings, they state a decentralisation of healthcare cannot resolve the regional disparities in the healthcare system of the country. The reason is high variability in the healthcare processes at a local level, the problems with staff recruitment, and so on. This outcome represents a challenge raised by the assumptions about the efficiency of the decentralised healthcare system.

The macroeconomic study explores a relationship between the health system resources and the economic conditions of the Organisation for Economic Co-operation and Development member countries, extracting the factors related to the introduction of the imaging diagnostic equipment in each observed country [12]. The authors classified the 29 countries into the four groups according to the medical environment and economic indices. The largest impact is kept by a number of hospital beds in a field of computed tomographs and magnetic resonance imaging scanners. Only one group of countries demonstrate the optimum conditions to introduce medical equipment. The authors call for a need to clarify the factors that would increase the likelihood of an efficient medical equipment deployment in each member state by examination of the high-growth medical equipment market.

An important criterion for purchase, deployment and usage of the health equipment in addition to an economic field is their increasing complexity. This is pointed out by the study, which states that it is important to understand the usefulness of the instruments and the methods for evaluation of the usability of the medical equipment, not only in the selection process, but also in the process of purchase [13].

A review of a relationship between the efficiency of the hospitals and the usage of computed tomographs and magnetic resonance imaging scanners is brought in more detail in the study [14]. The authors examine the 131 hospitals in China in the period from the year 2009 to the year 2013. The study did not differentiate appropriate and inappropriate usage of computed tomographs and magnetic resonance imaging scanners. The results show that higher usage of this equipment is associated with higher inefficiency of the healthcare facilities. Such findings have important political implications for a health system reform in China.

On the most important issues of management of the medical equipment usage in the public health sector in Benin is focused another study [15]. Between the year 2008 and the year 2010, they conducted the two surveys with the key actors in the healthcare system. The 377 questionnaires and the 259 interviews are involved in the first research, whilst the group interviews are engaged in the second round. From the results of the surveys, the authors conclude that the underlying problems are related to corruption at different levels of management and maintenance. The authors call for the necessity of a development of the policy and management tools to regulate the deployment of medical equipment, a usage of reference price lists in the public procurement of these devices, a development of the policy tools to regulate financial resources for the life cycle costs of technology, and the similar control mechanisms. It is also important to create new action plans for healthcare technology management and to develop new related policies.

Assessment of the availability of the healthcare technologies in Nepal is carried out in the study with a definition of the factors leading to optimum availability of the healthcare service and addressing of the problem areas [16]. Although the private health sector is relatively well developed in the country and achieves higher efficiency than the state system, the state system provides better healthcare for patients after injuries. The study highlights a number of categorised problems, the elimination of which would make healthcare more affordable and sustainable. For this, it is necessary to create a system of more efficient planning and management. In relation with the pressures to increase planning and management efficiency in the healthcare facilities, a research interest on finding optimum solutions is a subject of the study with the seven hospitals localised in the Santa Catarine state in the Federative Republic of Brazil [17]. There are surgical facilities, and the research is focused on cardiovascular surgery, which has a high degree of technological diversity due to their specialisation. The authors apply a multicriteria decision aid methodology, which is employed to set the appropriate benchmarks and to identify opportunities for improvement. They emphasise the importance of clinical engineering. At the same time, they call for the need for multidisciplinary engagement of experts in addressing the issue.

This is also in line with the findings of the study, which is interested in the research of the healthcare facilities in the Republic of Turkey [18]. These authors point out the importance in introduction and usage of the medical equipment in the process of a reduction of healthcare costs and an increase of healthcare quality. The question of finding optimum ways and addressing the key issues of the availability and efficiency of the usage of modern healthcare technologies is at the forefront. These issues are examined not only at a national level but also at a regional level.

The experts also address an issue of the availability and the economic sustainability of local public healthcare facilities, and they look for ways to achieve higher quality healthcare through the usage of the technological innovations even in smaller regional healthcare facilities [11].

There are the authors who see a problem in the incorrect specification of user requirements for the medical equipment [19], the others see an issue in their final stages—in an assessment of the organisational impact of the medical equipment usage [20].

There are also the studies calling for the importance of the health technology assessment and to take into an account the nonclinical areas [21,22,23,24,25,26]. The authors state that the focus is laid on the aspects related to the medical equipment, but an organisational impact remains unexplored in recent years. Many of them can have a critical impact on the healthcare organisation, and they are able to influence decision-making and regulatory processes significantly. This provides an opportunity to find efficient ways to rationalise the usage of the medical equipment—for instance through outsourcing.

The interesting incentives to improve efficiency in this area are also provided by the study [27]. The authors examine the research studies on this issue from a number of the databases with a large proportion of the studies with the mathematical models, the empirical studies and the conceptual documents. The authors point out that research about outsourcing in the usage of the medical equipment, which is called equipment maintenance processes by them, is still in its early stages and therefore, it is necessary to call for further empirical studies and to explore ways to increase efficiency in the usage of the medical equipment.

## 3. Data and Methodology

The Data and Methodology section offers a view on the data applied in the analysis and the methodology employed to carry out this analysis with the given data.

The data applied in the analysis come from the National Health Information Center of the Slovak Republic and from the Statistical Office of the Slovak Republic. The data about the medical equipment are provided by the first institution, and it is involved in the annual report of the National Health Information Center about the medical equipment in the healthcare facilities. This analysis takes into consideration the numbers of the two medical equipment—the most expensive ones that serve in practice. These are computed tomographs and magnetic resonance imaging scanners.

The data about the population are established on the year-end basis, that is, it is based on the situation valid on the 31st December of the individual year. The particular original table is marked om7009rr in Datacube [28], which serves as the main online database of the Statistical Office of the Slovak Republic.

Alongside the scientific methods, a part of the methodology is created also by the territorial understanding of the explored area.

The cluster analysis is employed to get a spatial classification of the individual districts of the Slovak Republic. As a hierarchical method of clustering, a hclust approach [29] is engaged, the pseudot2 approach [30] is selected to determine the numbers of clusters for each case of the analysis [31].

The pseudot2 approach value is calculated as follows:

$$P=\frac{\sum_{j=1}^{q}\sum_{g\in C_{x}}\left(v_{g}-c_{j}\right){\left(v_{g}-c_{j}\right)}^{T}-\sum_{k=1}^{q}\sum_{h\in C_{y}}\left(v_{h}-c_{k}\right){\left(v_{h}-c_{k}\right)}^{T}-\sum_{l=1}^{q}\sum_{i\in C_{z}}\left(v_{i}-c_{l}\right){\left(v_{i}-c_{l}\right)}^{T}}{\frac{\sum_{k=1}^{q}\sum_{h\in C_{y}}\left(v_{h}-c_{k}\right){\left(v_{h}-c_{k}\right)}^{T}+\sum_{l=1}^{q}\sum_{i\in C_{z}}\left(v_{i}-c_{l}\right){\left(v_{i}-c_{l}\right)}^{T}}{n_{y}+n_{z}-2}}$$

where the individual variables express:

− *P*—pseudot2 value,
− *j*—a rank of the cluster in the first computation,
− *q*—a number of the clusters,
− *g*—a rank of the district in the *C_x_* cluster,
− *v_g_*—a vector of the observation of the g-th district in the *C_x_* cluster,
− *c_j_*—a centroid of the first computation,
− *k*—a rank of the cluster in the second computation,
− *h*—a rank of the district in the *C_y_* cluster,
− *v_h_*—a vector of the observation of the h-th district in the *C_y_* cluster,
− *c_k_*—a centroid of the second computation,
− *l*—a rank of the cluster in the third computation,
− *i*—a rank of the district in the *C_z_* cluster,
− *v_i_*—a vector of the observation of the i-th district in the *C_z_* cluster,
− *c_l_*—a centroid of the third computation,
− *n_y_*—a number of the districts in the *C_y_* cluster,
− *n_z_*—a number of the districts in the *C_z_* cluster.

Similarity is quantified by the Euclidean distance that is computed through the following formula:

$$ED\left(d_{1},d_{2}\right)=\sqrt{{\left(x_{1}-x_{2}\right)}^{2}+{\left(y_{1}-y_{2}\right)}^{2}}$$

where the comprised variables mean:

− *d*_1_—the first district,
− *d*_2_—the second district,
− *ED*(*d*_1_, *d*_2_)—the mutual Euclidean distance of the *d*_1_ district and the *d*_2_ district,
− *x*_1_—the *x* coordinate of the *d*_1_ district,
− *x*_2_—the *x* coordinate of the *d*_2_ district,
− *y*_1_—the *y* coordinate of the *d*_1_ district,
− *y*_2_—the *y* coordinate of the *d*_2_ district.

The essential explanation of the previously mentioned formula expresses similarity quantified in units of the Euclidean distance. The smaller distance between the districts is, the more similar they are. The original input data are normalised in order to get the applied dimensions comparable within the analysis.

The fundamental legend for heat map says the darker colour is, the more dissimilar pair of the district it is and vice versa. Also, for the standard maps, the legend says that shade of the colour rises with an increasing number of the illustrated dimension.

### 3.1. Preventable Mortality

Preventable mortality is one part of a concept of avoidable mortality. It represents the specific diagnoses which mortality in the terms of causing death can be considered preventable from. Its structure is defined according to the document of Eurostat—the statistical office of the European Union [32]. Defining this cause of death could be also in other way because of usage of another list of preventable mortality diagnoses. For instance, the Office for National Statistics of the United Kingdom of Great Britain and Northern Ireland has its own list [33].

A whole set of the preventable mortality diagnoses is divided into the following 27 groups of the diagnoses:

− tuberculosis—A15 to A19, B90,
− hepatitis C—B17, B18,
− human immunodeficiency virus and acquired immunodeficiency syndrome—B20 to B24,
− malignant neoplasm of lip, oral cavity and pharynx—C00 to C14,
− malignant neoplasm of oesophagus—C15,
− malignant neoplasm of stomach—C16,
− malignant neoplasm of colon and rectum—C18 to C21,
− malignant neoplasm of liver—C22,
− malignant neoplasm of trachea, bronchus and lung—C33, C34,
− malignant melanoma of skin—C43,
− mesothelioma—C45,
− malignant neoplasm of breast—C50,
− malignant neoplasm of cervix uteri—C53,
− diabetes mellitus—E10 to E14,
− alcohol related diseases excluding external causes—F10, G31, G62, I42, K29, K70, K73, K74, K86,
− illicit drug use disorders—F11 to F19,
− ischaemic heart disease—I20 to I25,
− deep vein thrombosis with pulmonary embolism—I26, I80, I82,
− aortic aneurysm and dissection—I71,
− influenza including swine flu—J09 to J11,
− pneumonia—J12 to J18,
− chronic obstructive pulmonary disorder—J40 to J44,
− transport accident—V01 to V99,
− accidental injury—W00 to W59,
− suicide and self-inflicted injury—X60 to X84, Y10 to Y34,
− homicide and assault—X85 to X99, Y00 to Y09,
− misadventures to patients during surgical and medical care—Y60 to Y69, Y83.

The groups of the diagnoses are sorted alphabetically according to their abbreviated designation for the first involved diagnosis.

There is to note that because of the data availability for the whole explored period, a few three-digit diagnoses are substituted by two-digit diagnoses. The B17.1 diagnosis is substituted by the B17 diagnosis, the B18.2 diagnosis by the B18 diagnosis, the G31.2 diagnosis by the G31 diagnosis, the G62.1 diagnosis by the G62 diagnosis, the I42.6 diagnosis by the I42 diagnosis, the K29.2 diagnosis by the K29 diagnosis, the K74.0 diagnosis with the K74.1 diagnosis, the K74.2 diagnosis, and the K74.6 diagnosis by the K74 diagnosis, the K86.0 diagnosis by the K86 diagnosis, the I80.1 diagnosis with the I80.2 diagnosis and the I80.3 diagnosis by the I80 diagnosis, and the I82.9 diagnosis by the I82 diagnosis. This alteration does not influence the analysis outcome in any considerable way.

### 3.2. Territorial Division

The elementary territorial division of the explored area is established on the Nomenclature of Territorial Units for Statistics, which serves as the primary geocode standard for Eurostat—the main statistical office of the European Union [34]. The analysis is based on the fourth level of this standard that is represented by the individual districts of the Slovak Republic.

The list of the abbreviated codes applied in the analysis and the inserted figures for the separate districts is subsequent:

− SK0101—the Bratislava I District,
− SK0102—the Bratislava II District,
− SK0103—the Bratislava III District,
− SK0104—the Bratislava IV District,
− SK0105—the Bratislava V District,
− SK0106—the Malacky District,
− SK0107—the Pezinok District,
− SK0108—the Senec District,
− SK0211—the Dunajská Streda District,
− SK0212—the Galanta District,
− SK0213—the Hlohovec District,
− SK0214—the Piešťany District,
− SK0215—the Senica District,
− SK0216—the Skalica District,
− SK0217—the Trnava District,
− SK0221—the Bánovce nad Bebravou District,
− SK0222—the Ilava District,
− SK0223—the Myjava District,
− SK0224—the Nové Mesto nad Váhom District,
− SK0225—the Partizánske District,
− SK0226—the Považská Bystrica District,
− SK0227—the Prievidza District,
− SK0228—the Púchov District,
− SK0229—the Trenčín District,
− SK0231—the Komárno District,
− SK0232—the Levice District,
− SK0233—the Nitra District,
− SK0234—the Nové Zámky District,
− SK0235—the Šaľa District,
− SK0236—the Topoľčany District,
− SK0237—the Zlaté Moravce District,
− SK0311—the Bytča District,
− SK0312—the Čadca District,
− SK0313—the Dolný Kubín District,
− SK0314—the Kysucké Nové Mesto District,
− SK0315—the Liptovský Mikuláš District,
− SK0316—the Martin District,
− SK0317—the Námestovo District,
− SK0318—the Ružomberok District,
− SK0319—the Turčianske Teplice District,
− SK031A—the Tvrdošín District,
− SK031B—the Žilina District,
− SK0321—the Banská Bystrica District,
− SK0322—the Banská Štiavnica District,
− SK0323—the Brezno District,
− SK0324—the Detva District,
− SK0325—the Krupina District,
− SK0326—the Lučenec District,
− SK0327—the Poltár District,
− SK0328—the Revúca District,
− SK0329—the Rimavská Sobota District,
− SK032A—the Veľký Krtíš District,
− SK032B—the Zvolen District,
− SK032C—the Žarnovica District,
− SK032D—the Žiar nad Hronom District,
− SK0411—the Bardejov District,
− SK0412—the Humenné District,
− SK0413—the Kežmarok District,
− SK0414—the Levoča District,
− SK0415—the Medzilaborce District,
− SK0416—the Poprad District,
− SK0417—the Prešov District,
− SK0418—the Sabinov District,
− SK0419—the Snina District,
− SK041A—the Stará Ľubovňa District,
− SK041B—the Stropkov District,
− SK041C—the Svidník District,
− SK041D—the Vranov nad Topľou District,
− SK0421—the Gelnica District,
− SK0422—the Košice I District,
− SK0423—the Košice II District,
− SK0424—the Košice III District,
− SK0425—the Košice IV District,
− SK0426—the Košice-okolie District,
− SK0427—the Michalovce District,
− SK0428—the Rožňava District,
− SK0429—the Sobrance District,
− SK042A—the Spišská Nová Ves District,
− SK042B—the Trebišov District.

### 3.3. Computation Technique

The whole computation, the diagrams and the maps are produced by the R software environment [29]. The rgdal package [35] serves to prepare the maps and the shape package creates the colour palette for the map. The heat maps are generated by the gplots package [36] and the cluster analysis is produced by the NbClust package [30]. The auxiliary work is supported by the data table package [37] and the raster package [38]. There is to note that all the figures and all the tables with the whole contents of the paper are elaborated by the authors.

## 4. Results

The whole analysis output can be divided into three sections. The first one is devoted to the analysis of the medical equipment distribution over all the districts of the Slovak Republic. The second one is aimed at the preventable mortality rate of the female sex population and the third one at the preventable mortality rate of the male sex. Each part consists of a description of a situation at the beginning of the explored period in the year 2008, a situation at the end of the explored period in the year 2015, and a situation throughout the whole examined period between these years. 

### 4.1. Medical Equipment Distribution

A pair of the two most expensive medical equipment is created by computed tomograph and magnetic resonance imaging scanner. The computed tomograph serves to provide a tomogram that is a computed tomography scan, or in the other words, a computerised axial tomography scan. The magnetic resonance imaging scanner makes available to picture an inner situation of a human body. Sometimes, it is referred to as the nuclear magnetic resonance imaging scanner or very rarely as the magnetic resonance tomograph.

**Figure 1:**
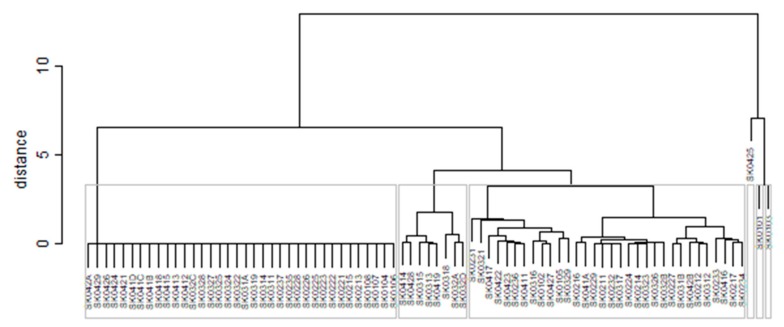
Medical equipment dendrogram of the districts in the year 2008. Source: own elaboration by the authors.

**Figure 2:**
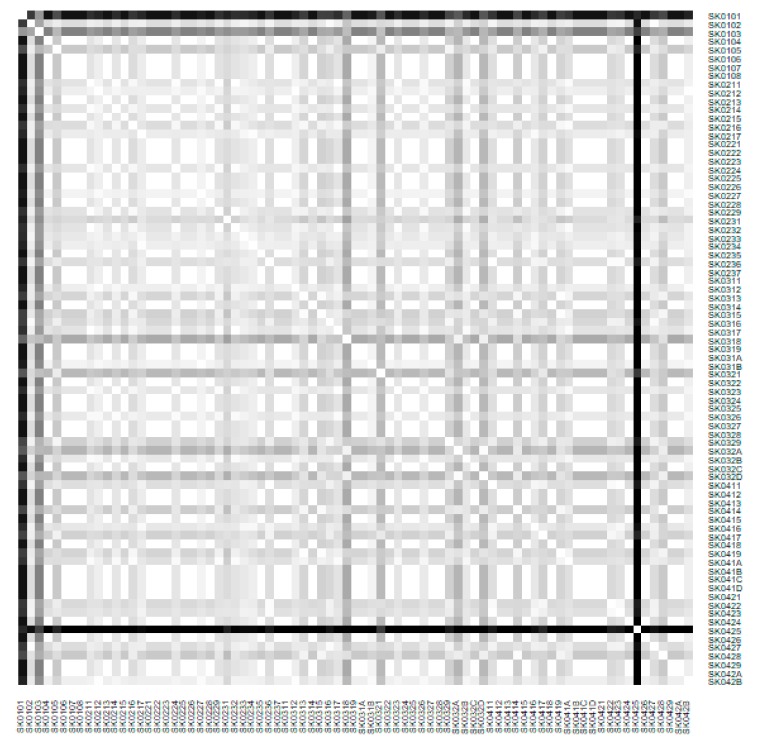
Medical equipment heat map of the districts in the year 2008. Source: own elaboration by the authors.

**Figure 3:**
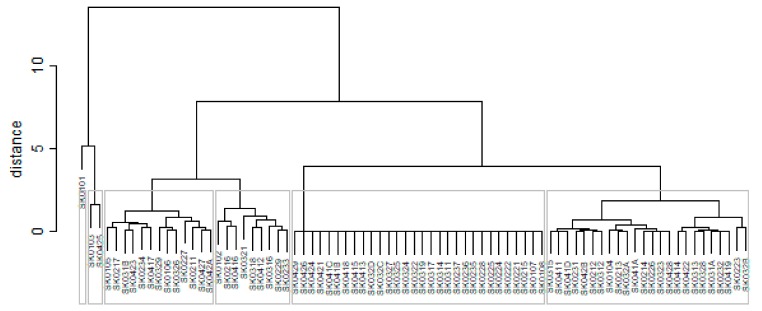
Medical equipment heat map of the districts in the year 2015. Source: own elaboration by the authors.

**Figure 4:**
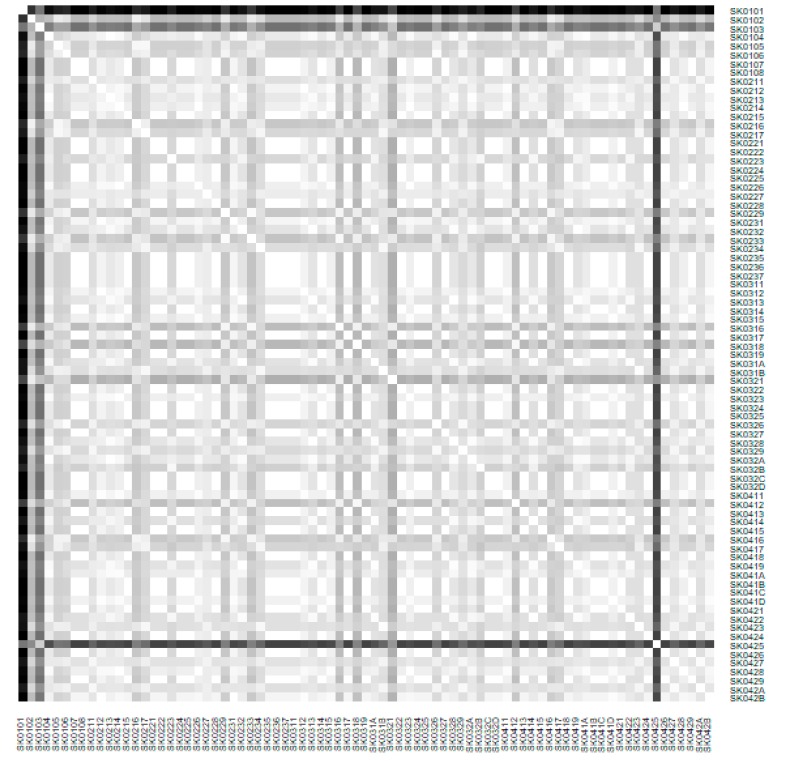
Medical equipment heat map of the districts in the year 2015. Source: own elaboration by the authors.

**Figure 5:**
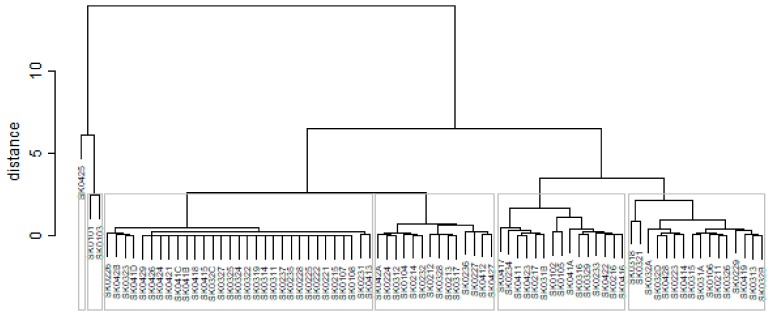
Medical equipment dendrogram of the districts in the whole explored period. Source: own elaboration by the authors.

**Figure 6:**
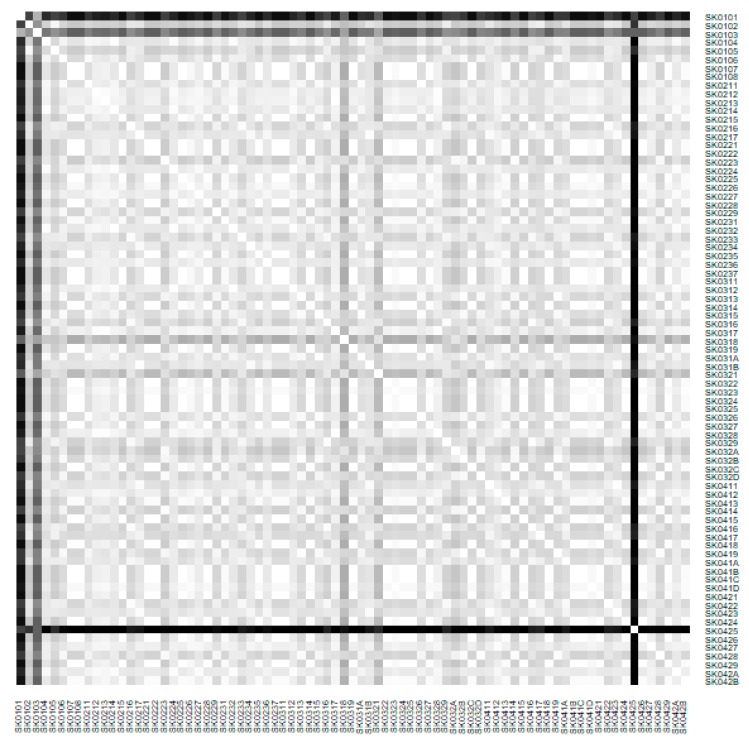
Medical equipment heat map of the districts in the whole explored period. Source: own elaboration by the authors.

As it is seen in Table 1, at the beginning of the observed period in the year 2008, a number of the computer tomographs is equal to 70 and until the end of the observed period in the year 2017, it raised to the level of 100 peaking the level of 103 in the year 2015. A similar development is recorded also for the magnetic resonance imaging scanners, as a number of 33 from the first year 2008 elevated to the level of 51 during the last year 2017. A positive trend is evident during the whole explored period. The subsequent figures—Figure 1 and Figure 2—demonstrate a situation of medical equipment distribution among the individual districts of the Slovak Republic at the beginning of the period in the year 2008. Similarly, a situation from the end of the explored period in the year 2015 is described in Figure 3 and Figure 4. A mean state related to the whole explored period is visualised by Figure 5 and Figure 6. The first figure pictures dendrogram that shows a distribution of the constructed clusters from the districts and their mutual Euclidean distance expressing similarity of the clusters. The latter one embodies heat map demonstrating mutual similarity through the Euclidean distance.

As the first point after the above-mentioned figures, there is to note that the distances at the level of 0 are caused by the fact that there is a lot of the districts with zero medical equipment. That is why these districts keep the same level of mutual distance and they are visualised on the same horizontal level. The districts with dark coloured striped possess the extreme positions against the rest of the Slovak Republic meaning that the Bratislava I District, the Bratislava II District, and the Košice IV District create a specific group from a territorial point of view regarding their mutual similarity. Hence, they are ready to apply a potential specific healthcare policy which would be different from the general regional policy applied for the whole Slovak Republic. Having a look at the situations at the beginning and at the end of the examined period, they are very similar to the previously mentioned conclusion. From a point of view of the interpretation, they are almost identical.

The following table shows the ten most extreme districts in a field of the medical equipment distribution for the beginning of the observed period in the year 2008, for the end of the observed period in the year 2015 and a mean situation of the whole observed period.

**Figure 7:**
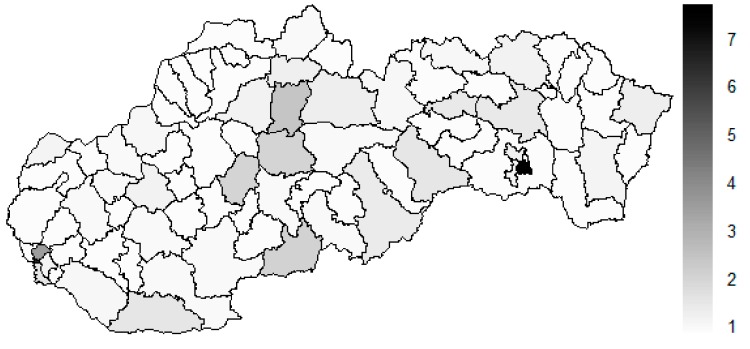
Medical equipment similarity map of the districts in the year 2008. Source: own elaboration by the authors.

**Figure 9:**
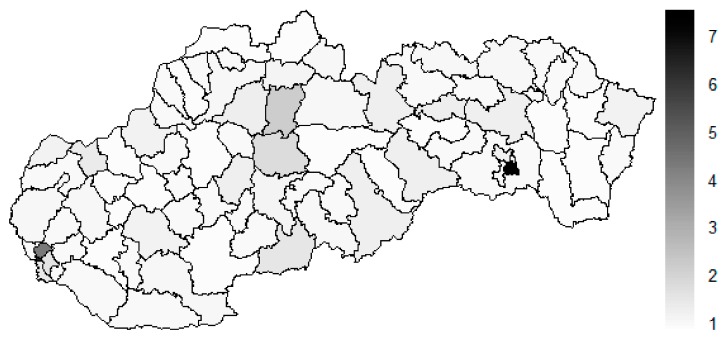
Medical equipment similarity map of the districts in the whole explored period. Source: own elaboration by the authors.

There is to note that the Euclidean distance shown in the above-mentioned table represents a mean value of the Euclidean distance of the given district in a mutual pair to every other one district of the Slovak Republic. The similarity values demonstrate how long is a Euclidean distance between the particular districts. Its mean value is calculated from all the distances assigned to the singular district. The individual clusters are apparently evident. As Table 2 demonstrates, the most extremely located districts are the Košice IV District, the Bratislava I District, and the Bratislava III District in the year 2008. It can be applied for both ends of the explored period, but with the Bratislava I District on the first position and the Košice IV District on the second position in the year 2015. This ranking of a mean situation for the whole analysed period is the same as for its beginning. A number of the constructed clusters is six, and it remains the same for all the three described cases. Their composition is very similar throughout the witnessed period. The mutual mean similarity decreases rapidly on the lower places of the above-mentioned rankings than on the first several places. The subsequent figures envisage a map visualisation of the districts based on the distances computed according to the distribution of the medical equipment—Figure 7 showing a situation in the year 2008, Figure 8 showing a situation in the year 2015, and Figure 9 showing a situation of the complete explored period.

As it is seen from the three previous figures, the examined medical equipment is localised very centrally in the terms of their concentration to the two largest cities of the Slovak Republic—Bratislava, which is the capital city, and Košice. Both cities are divided into several districts because of its level of population. Bratislava is created by the five districts, and two of them are highlighted—the Bratislava I District and the Bratislava III District. Košice is formed by the four districts, and its surrounding rural area is also represented by the own district. The Košice IV District is the featured district because of its large number of the equipment. A more illustrative view is offered by a successive triplet of the figures where the extreme districts are omitted from the legend scale of the maps.

**Figure 10:**
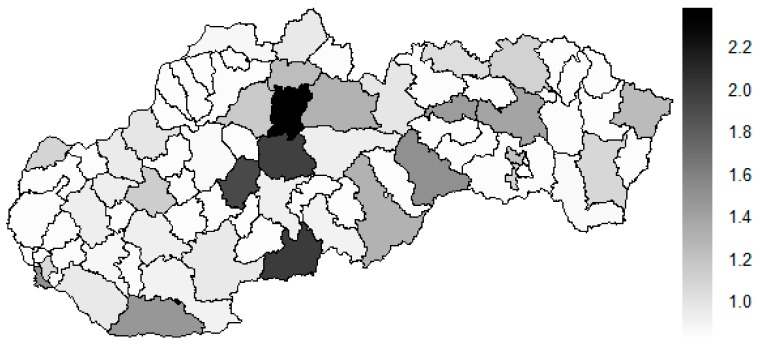
Modified medical equipment similarity map of the districts in the year 2008. Source: own elaboration by the authors.

**Figure 11:**
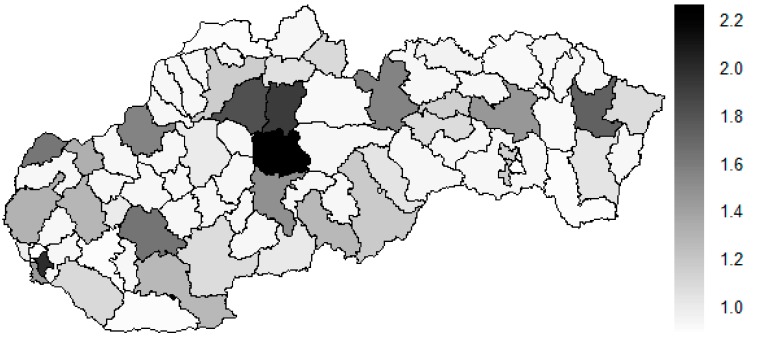
Modified medical equipment similarity map of the districts in the year 2015. Source: own elaboration by the authors.

**Figure 12:**
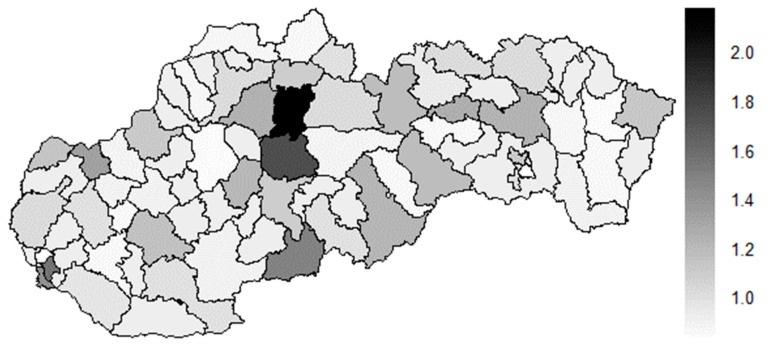
Modified medical equipment similarity map of the districts in the whole explored period. Source: own elaboration by the authors.

In the previously mentioned three maps visualised on Figure 10, Figure 11 and Figure 12, a maximum value to be displayed is determined by the fourth largest Euclidean distance in the appropriate vector of the district’s values. Due to this exclusion of the first three largest values, the districts assigned by them are filled by the lightest colour shade, that is, white colour. This step enables to see the similarities of the districts on the remaining positions in a better way. A very dark area in the middle of the Slovak Republic is created by the Banská Bystrica District, the Ružomberok District, and the Martin District. A poorly developed situation is seen in the Veľký Krtíš District, where a significant fall is recorded throughout the observed period.

### 4.2. Female Sex Preventable Mortality

**Figure 13:**
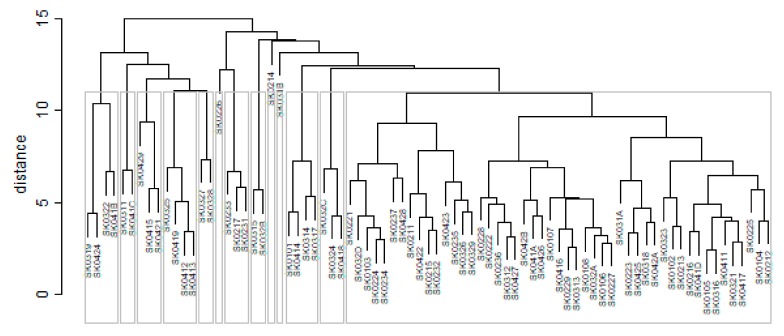
Preventable mortality dendrogram of the districts for the female sex in the year 2008. Source: own elaboration by the authors.

**Figure 14:**
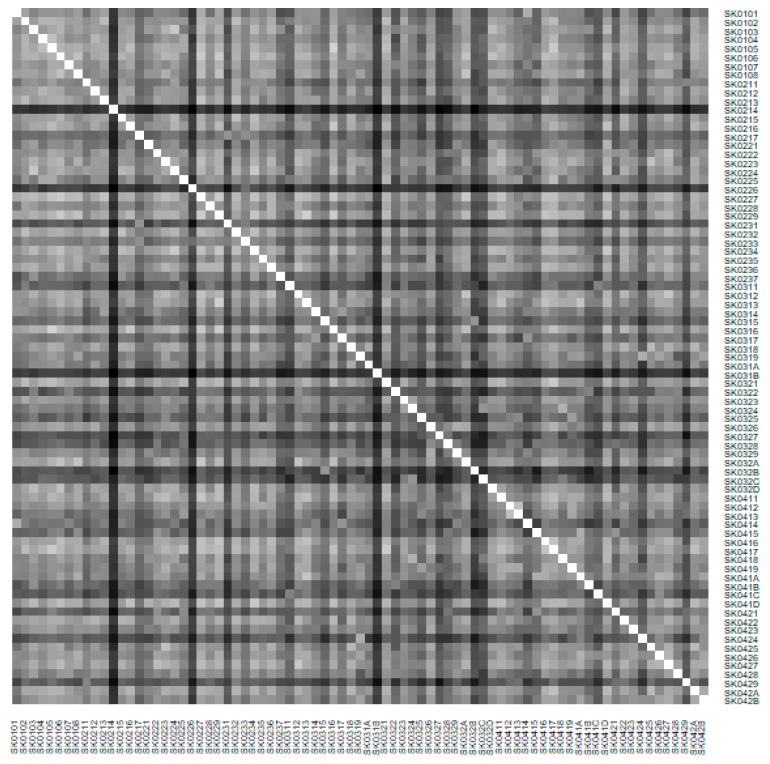
Preventable mortality heat map of the districts for the female sex in the year 2008. Source: own elaboration by the authors.

**Figure 15:**
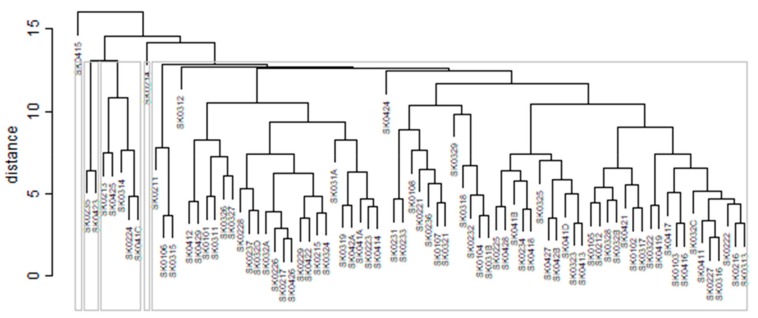
Preventable mortality dendrogram of the Districts for the female sex in the year 2015. Source: own elaboration by the authors.

**Figure 16:**
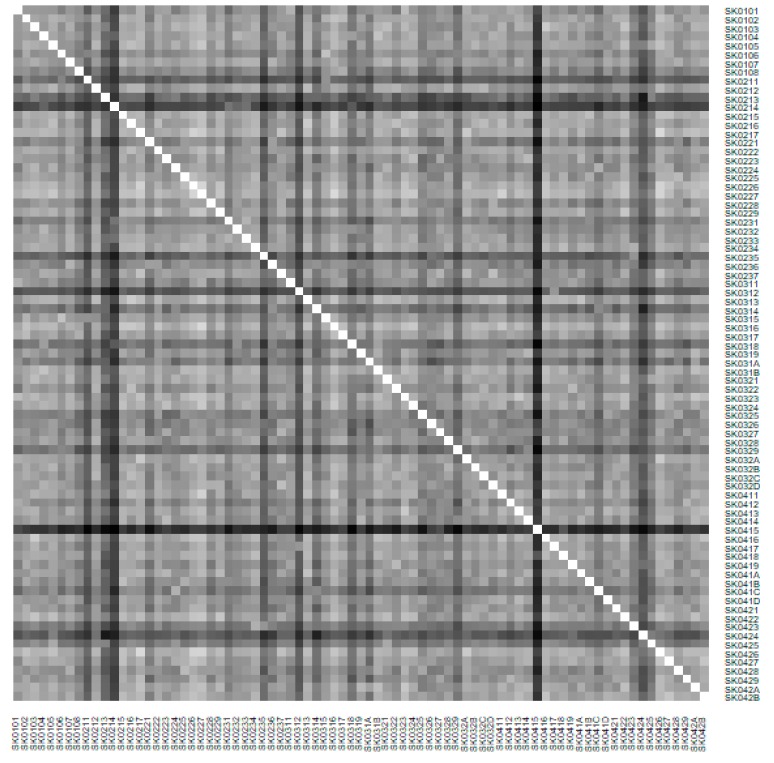
Preventable mortality heat map of the districts for the female sex in the year 2015. Source: own elaboration by the authors.

**Figure 17:**
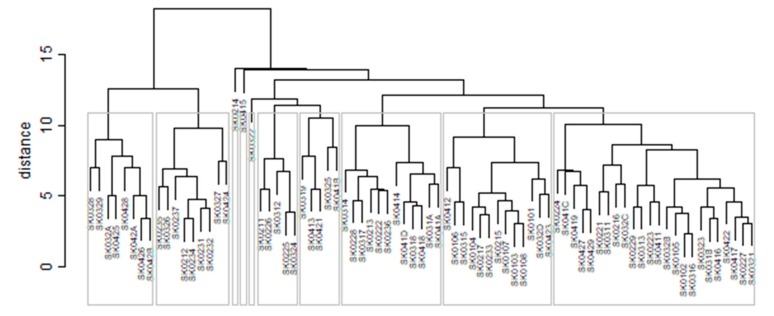
Preventable mortality dendrogram of the districts for the female sex in the whole explored period. Source: own elaboration by the authors.

**Figure 18:**
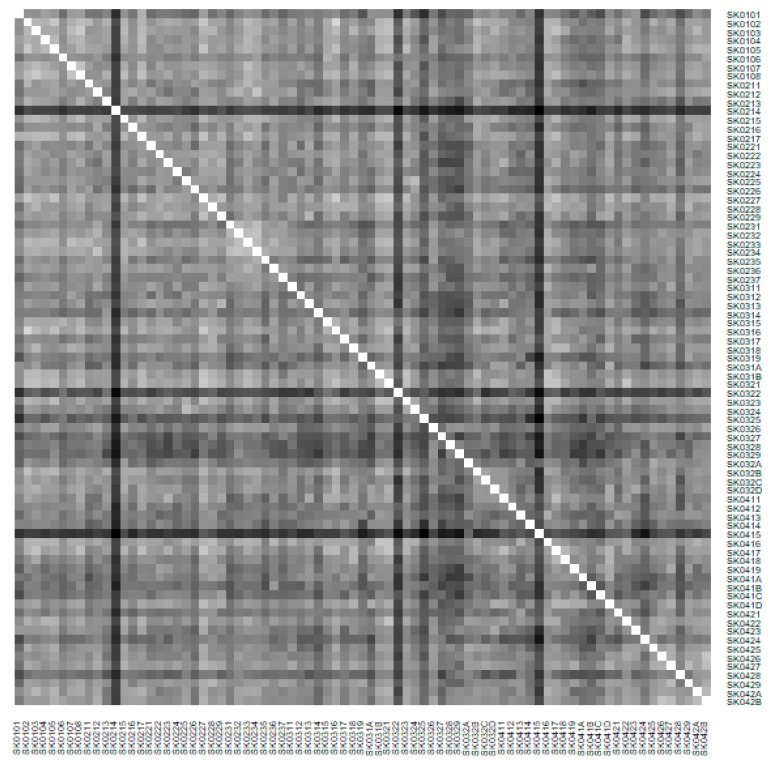
Preventable mortality heat map of the districts for the female sex in the whole explored period. Source: own elaboration by the authors.

The subsequent diagrams illustrated on Figure 13, Figure 14, Figure 15, Figure 16, Figure 17 and Figure 18 bear the same structure as the first six figures, although they are aimed at the preventable mortality diagnoses of the female sex.

As it is seen from a triplet of the previously mentioned heat maps, the preventable mortality similarity is considerably various among the districts of the Slovak Republic for the female sex. This implies a peculiar role of the regional healthcare policy because it should be tailor-made for the individual areas of the country in order to be as precise and as efficient as it is possible. There are visible the outliers which a specific policy should be applied on in order to get them into the regime of a remaining area of the country, because the more districts possess the outlying positions, the more complicated it is. Hence, it is determined which regional healthcare policy suits for them best. At the beginning of the explored period, the Piešťany District, the Považská Bystrica District and the Žilina District perform as the significant outliers, whilst the first one along with the Medzilaborce District stay at the extreme positions at the end of the examined period too. Unsurprisingly, both these districts keep their outlying position throughout the whole observed period and the Banská Štiavnica District with them too.

The subsequent table shows the ten most extreme districts in a field of the female sex preventable mortality for the beginning of the explored period in the year 2008, for the end of the explored period in the year 2015 and a mean situation of the whole explored period.

**Figure 19:**
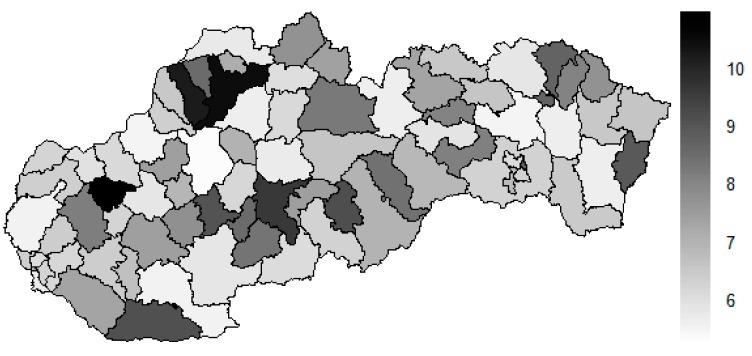
Preventable mortality similarity map of the districts for the female sex in the year 2008. Source: own elaboration by the authors.

**Figure 20:**
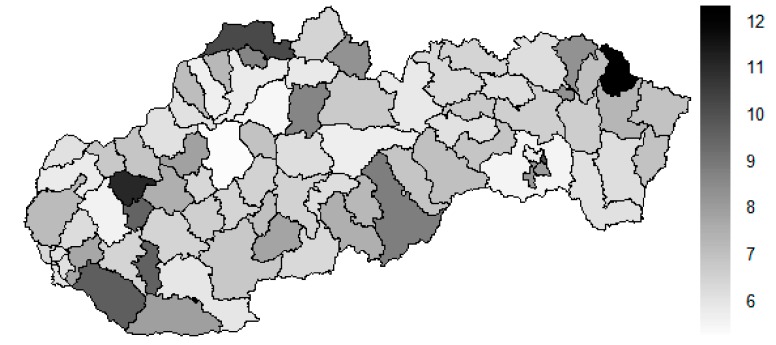
Preventable mortality similarity map of the districts for the female sex in the year 2015. Source: own elaboration by the authors.

**Figure 21:**
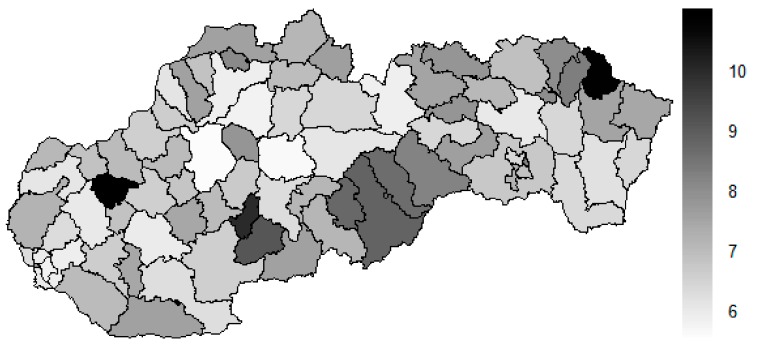
Preventable mortality similarity map of the districts for the female sex in the whole explored period. Source: own elaboration by the authors.

There is to note that the Euclidean distance shown in the above-mentioned table represents a mean value of the Euclidean distance of the given district in a mutual pair to every other one district of the Slovak Republic meaning it expresses a potential similarity to each district. As Table 3 states, the most extreme triplet of the districts at the beginning of the explored period is created by the Piešťany District, the Žilina District, and the Považská Bystrica District. During this period a situation of the female sex changed noticeably as the Medzilaborce District, the Piešťany District, and the Čadca District stand at the top with the most extreme values. In a case of the whole period, besides the Piešťany District and the Medzilaborce District, the Banská Štiavnica District holds the third position. Generally, the whole period seems to be raging. The following figures visualise a geographical view of the Slovak Republic districts according to the distances calculated regarding the female sex preventable mortality data—the beginning of the explored period is visualised in Figure 19, the end of this period on Figure 20, and the whole period on Figure 21.

The above-mentioned maps appropriately illustrate the development of the similarity of the districts in a field of the standardised mortality rate of the preventable diagnoses for the female sex. A considerable shift to the most extreme values is seen in a case of the Medzilaborce District and the Žilina District. Also, the Banská Štiavnica District and the Čadca District are on a move to the maximum values. The Dunajská Streda District, the Liptovský Mikuláš, the Šaľa District, and the Zvolen District are the ones where a similar movement is visible. An opposite shift is seen in the Trnava District and partially in the Košice-okolie District too.

### 4.3. Male Sex Preventable Mortality

**Figure 22:**
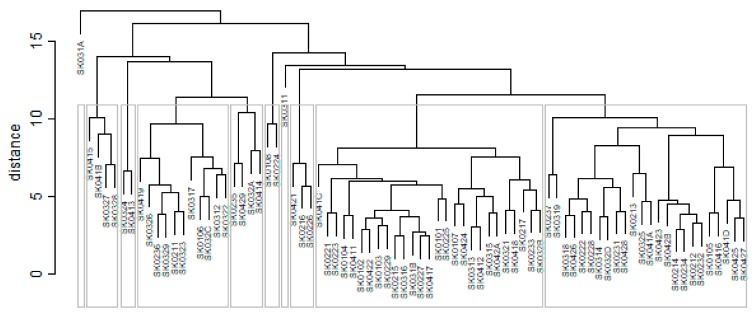
Preventable mortality dendrogram of the districts for the male sex in the year 2008. Source: own elaboration by the authors.

**Figure 23:**
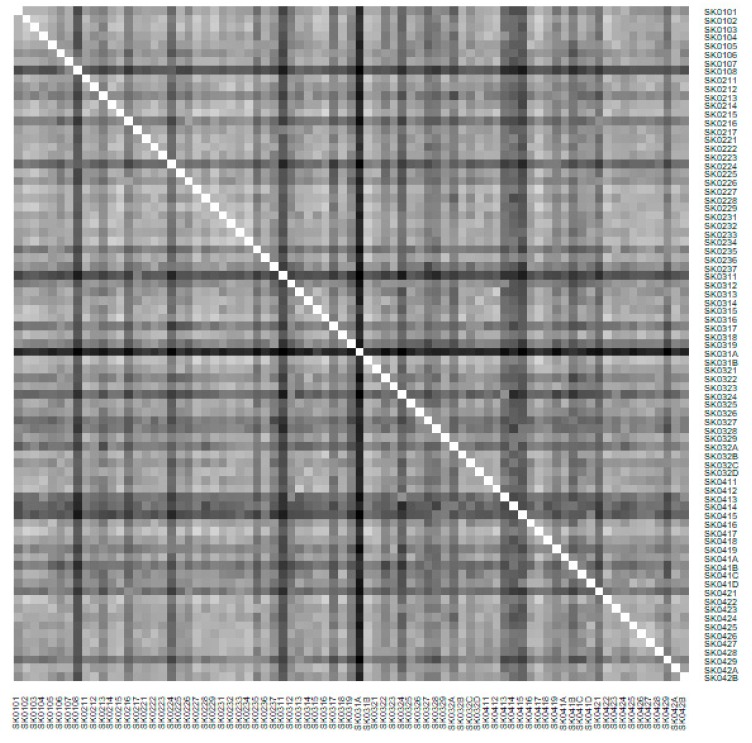
Preventable mortality heat map of the districts for the male sex in the year 2008. Source: own elaboration by the authors.

**Figure 24:**
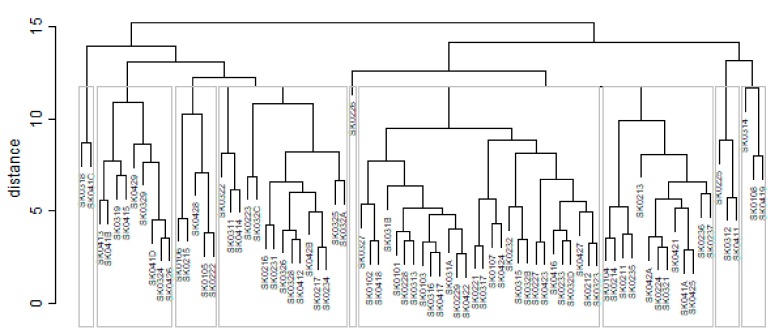
Preventable mortality dendrogram of the districts for the male sex in the year 2015. Source: own elaboration by the authors.

**Figure 25:**
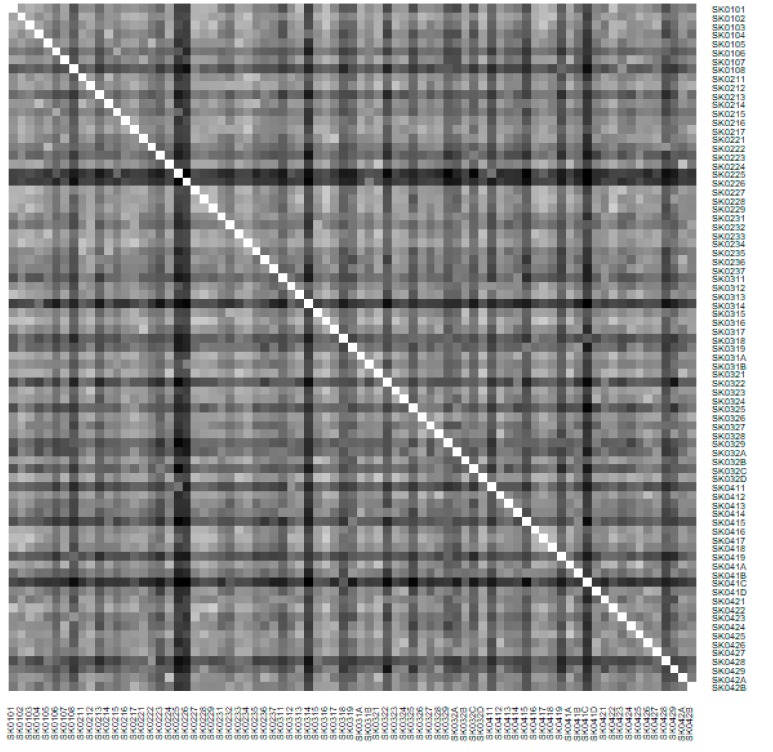
Preventable mortality heat map of the districts for the male sex in the year 2015. Source: own elaboration by the authors.

**Figure 26:**
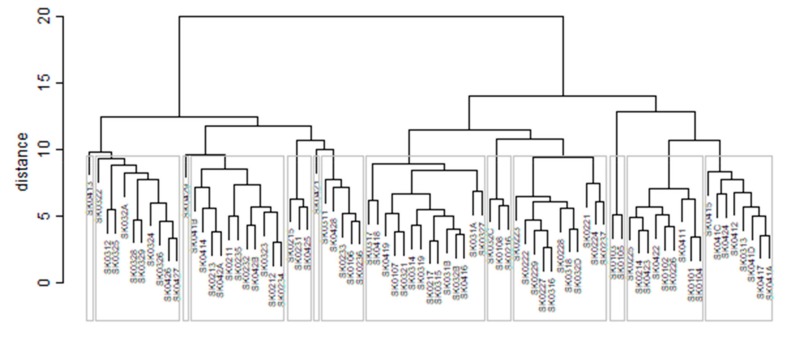
Preventable mortality dendrogram of the districts for the male sex in the whole explored period. Source: own elaboration by the authors.

**Figure 27:**
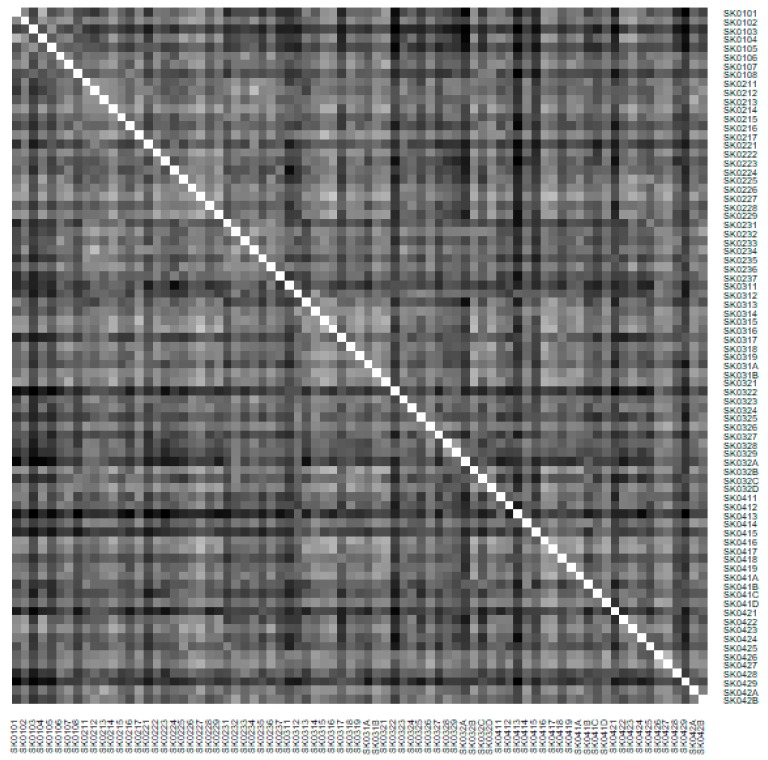
Preventable mortality heat map of the districts for the male sex in the whole explored period. Source: own elaboration by the authors.

The subsequent diagrams visualised on Figure 22, Figure 23, Figure 24, Figure 25, Figure 26 and Figure 27 keep the same structure as the previous six ones, regarding their aiming at the preventable mortality diagnoses of the male sex.

The outcome of the similarity heat maps is for the male sex different absolutely as for the female sex. The Tvrdošín District is the only district with an outlier attribute observing the beginning of the explored period. There are the four districts located at the extreme position at the end of this period—the Partizánske District, the Považská Bystrica District, the Kysucké Nové Mesto District, and the Svidník District. A look at the heat map of the whole explored period offers the most varying view certainly. None of the districts can be selected as the proper outlier.

The succeeding table shows the ten most extreme districts in a field of the male sex preventable mortality for the beginning of the explored period in the year 2008, for the end of the explored period in the year 2015 and a mean situation of the whole explored period.

The same legend as for Table 3 is valid also for Table 4.

**Figure 28:**
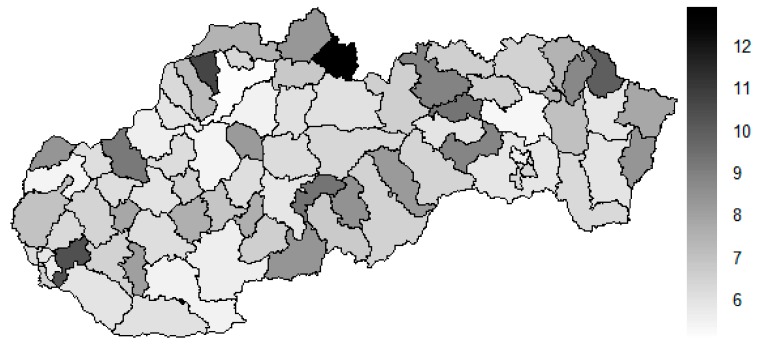
Preventable mortality similarity map of the districts for the male sex in the year 2008. Source: own elaboration by the authors.

**Figure 29:**
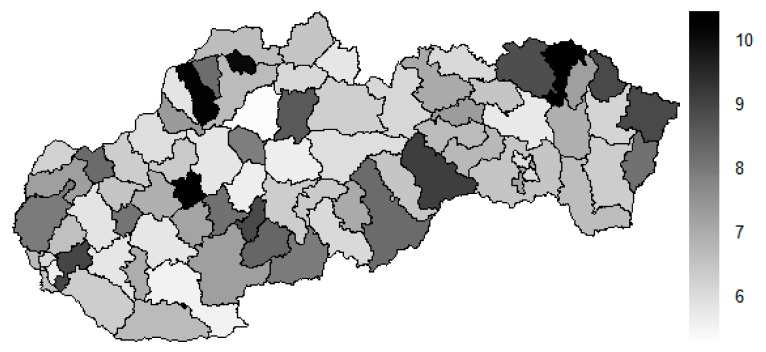
Preventable mortality similarity map of the districts for the male sex in the year 2015. Source: own elaboration by the authors.

**Figure 30:**
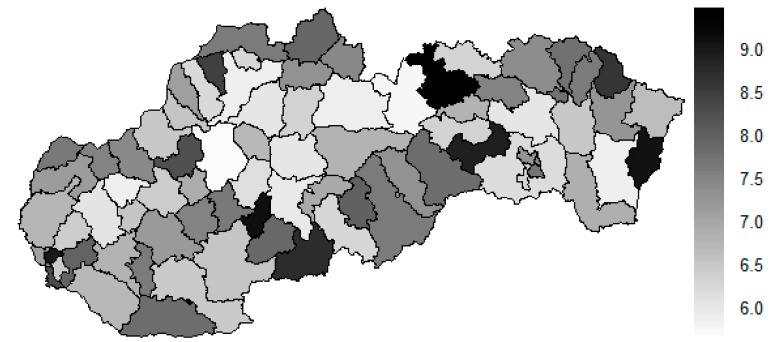
Preventable mortality similarity map of the districts for the male sex in the whole explored period. Source: own elaboration by the authors.

As Table 4 expresses, the Tvrdošín District, the Bytča District, and the Senec District represent the most extreme districts in the year 2008, whilst in the year 2015, it is an absolutely different triplet—the Partizánske District, the Svidník District, and the Považská Bystrica District. Also, other different districts represent the most extreme triplet of the whole period – the Kežmarok District, the Banská Štiavnica District, and the Sobance District. The subsequent figures picture a geographical outlook of the Slovak Republic districts based on the similarity computed through the Euclidean distance for the male sex preventable mortality rate—Figure 28 illustrating the beginning of the examined period, Figure 29 the end of this period, and Figure 30 the whole period.

A situation is categorically different for the male sex as it is for the female sex. The following districts have moved to the extreme values—the Banská Štiavnica District, the Kysucké Nové Mesto District, the Malacky District, the Myjava District, the Partizánske District, the Považská Bystrica District, the Rožňava District, the Ružomberok District, the Rožňava District, and the Svidník District. Besides these districts, also the Bratislava III District, the Bratislava V District, the Košice I District, and the Košice IV District have got their distances higher. An opposite situation has occurred only the Tvrdošín District where its extreme value has been decreased. Overall, there are districts with an increasing number of distances, that is, becoming more dissimilar with their surroundings than vice versa.

## 5. Discussion

A focus of this research is not only on the avoidable mortality and within it on the preventable mortality, which is influenced by the availability of healthcare. It also includes the availability of medical equipment. Looking at the results of the analysis, the interesting findings can be seen. The number of computer tomographs and magnetic resonance imaging scanners in the individual districts has been increasing since 2008, with the exception of stagnation of a number of magnetic resonance imaging scanner in the last three years. Although there is a noticeable increase in the number of the explored medical equipment, its distribution within the districts is considerably uneven. These findings confirm the result of the cluster analysis. The extreme values in the terms of its distribution are achieved by the Košice IV District at the level of 7.50630 units of Euclidean distance, the Bratislava I District at 6.56821, and the Bratislava III District at 4.35243 throughout the whole explored period. This is related to the fact that there are large university hospitals and the specialised healthcare facilities in these districts who’s diagnostic and treatment processes are associated with the usage of this medical equipment. The outcome of the analysis of the preventable mortality rate during the same period point to the different regional distribution than the computed one regarding the medical equipment, both for the female sex and the male sex. The preventable mortality rate has developed differently within the individual districts throughout the observed period. The highest values are reached by the Piešťany District peaking at the level of 10.97969 units of Eucliden distance, the Medzilaborce District at 10.92476, and the Banská Štiavnica District at 9.99689. Regarding the sexes, the results of the preventable mortality rate are more heterogeneous in the male sex than in the female sex within the individual districts. There is also a complete alteration in the extremely ranked districts during the explored period. From a statistical point of view, the averaging of the data for the whole period is also an affecting factor. Development of the preventable mortality rate values has been uneven not only in a territorial point of view, but also from a time aspect. In many districts with a recorded increase in the mortality rate, there is a decline in several years, and vice versa. Among the extreme districts with the highest recorded preventable mortality rate, the Kežmarok District is placed on the first position peaking at 9.44088 units of Euclidean distance, the Banská Štiavnica District at 9.10647, and the Sobrance District at 9.09178. The following table offers a view on the districts, where there is an absolute lack of computed tomographs and magnetic resonance imaging scanners.

As it is seen in Table 5, the districts with no explored medical equipment bear the high levels of the preventable mortality rate. The mean preventable mortality rate is calculated for the whole observed period. The final column represents an order of the district according to the mean preventable mortality rate among all the districts of the Slovak Republic. Though, a majority of the districts mentioned here comes from the lower half of an ordered list. It is a clear sign of an inappropriate state of health held by the population of the districts where no computed tomograph and magnetic resonance imaging scanner is available.

The districts with at least one piece of the examined medical equipment show the lower levels of the preventable mortality rate in general. Moreover, Table 6 shows another interesting fact too. Although, the six worst districts possess any of the explored medical equipment. However, there are other possible causes of this state too. By a comparison of the mean preventable mortality rate values, it is easily demonstrated that a presence of the diagnostic medical equipment influences this rate, because the territory of the districts without the examined medical equipment bears the preventable mortality rate at the level of 745.3945 and this value is remarkably higher than the level assigned to the remaining part of the country without the mentioned medical equipment standing at 700.8709.

Uneven distribution of the medical equipment over the country could also affect the regional disparities in a mortality rate development. In the period when the availability of computed tomography and magnetic resonance imaging is very limited, the introduction of a finding that an increase in computed tomographs leads to the regional differences in its deployment and usage is found in the study [6]. Already during this period, they stress the need to create regional computed tomography system for their optimal regional distribution. The reason is to achieve lower total costs within such a system.

There is a clear tendency to disseminate the outputs towards confronting their abundance with the quantifiable economic parameters also in many other research studies aimed at exploring the regional differences in the distribution of medical equipment. These parameters are examined by the several authors in the early stages, for instance in the procurement of the medical equipment [19], as well as in the final stages, for instance in the assessment of economic efficiency of their usage [39,40].

However, research studies that confront the distribution of the medical equipment with the development of the mortality rate are absent. Looking at the core of preventable mortality, one of the possibilities of its elimination is higher availability of healthcare and a higher quality of its provision. This is also ensured to a greater extent by a higher level of the medical equipment usage. Therefore, it is important to look for a causal relationship between the condition and development of mortality and the distribution of the medical equipment. A high rate of the medical equipment usage in a given location should correspond to a low level of the preventable mortality rate and vice versa. If these relationships are not confirmed, it is important to examine other factors that cause such association. Health literacy and health behaviour, as well as a wide range of the socio-economic factors related to economic and social inequalities in the regions, are also highlighted. An interesting research trajectory would be to investigate the regional differences in the distribution of the medical equipment and preventable mortality within the least developed districts, which are also defined by the legal norm [41]. It is expected that the status of the least developed regions can also be related to a number of the healthcare facilities and the limited choice of healthcare services which are provided by them.

There are several limitations to this study. Mainly, they are related to the source database. The data from the National Health Information Center on the medical equipment are obtained only at a district level in the most detail without the possibility of assigning them to the specific healthcare facility in the particular district of the Slovak Republic.

Another limitation of the study is the type of medical equipment that is analysed. Although the numerous studies declare an influence of computed tomograph and magnetic resonance imaging scanner numbers on the condition and development of mortality of serious diagnoses, their number in the individual districts is recorded only in units, not at least in tens of units. This seriously limits a choice of an appropriate methodological procedure to investigate causal relationships between the numbers of this medical equipment and mortality, as for instance an increase by one unit from a basement at the level of one unit means a relative increase by 100%.

Nevertheless, this study bears the significant dissemination outputs for health policy interventions, for instance, to draft regional healthcare plans for a deployment of the computed tomograph and magnetic resonance imaging scanner, especially in locations with the high preventable mortality rate. Specific prevention programmes would play an important role in this field. They should be a part of the policies that affect the regulation of a medical equipment deployment as well as the regulation of the financial resources necessary to maintain it. It is also important to create new action plans for healthcare technology management and health technology assessment. This is related to the fact that in addition to the previously preferred assessment techniques of health-related aspects of the medical equipment, nonclinical areas and the impact of medical devices on organizational processes have recently begun to be measured. These dimensions are also extremely important for improving the quality of healthcare, which is immediately reflected in the level and structure of morbidity and mortality. These consistent facts reaffirm the importance of health technology distribution assessment in relation to mortality reflecting the quality and availability of healthcare in the region.

The limitations lie in a field of the data set partially. The main ambition of the analysis is to investigate the relationship between the medical equipment along with its localisation in the particular healthcare facilities over the districts of the Slovak Republic and the standardised mortality rate of the preventable diagnoses. However, such detailed data are not available from the National Health Information Centre. Another limitation is a lack of studies that declare and assess the level of the benefits of the diagnostic processes using the examined medical equipment. Hence, also a lack the studies to reveal a relationship between the mentioned diagnostic processes and the particular preventable mortality diagnoses. It would be beneficial to add also further information about the medical equipment. In general, use of the observed types of the medical equipment is a very demanding issue. A missing interconnection of the morbidity database and the mortality database performs as a key factor. There are no analyses, which use of the diagnostic medical equipment is investigated in relation to early reveal of the potential diseases in. A possible field to research is offered by the inspection of the various stages of the diseases with a concentration on the primary phase of the disease outbreak. This study could put pressure on the development of the above-mentioned healthcare database and their modification for their use in the analytical processes that could help to improve health policy plans and hence, it would be valuable to the international and national benchmark.

## 6. Conclusions

The outcome of this analysis is offered mostly in a visualisation form. This makes it possible to better assess the relationship that is the primary subject of the research. The main aim of the study is to investigate the relationship between the spatial distribution of the selected medical equipment and preventable mortality in the regions of the Slovak Republic in the period beginning in the year 2008 and ending in the year 2015. The 27 groups of the preventable mortality diagnoses and the two types of the medical equipment, where computed tomograph and magnetic resonance imaging scanner belong, are examined within all the districts of the country. After exploration of a wide range of the international research studies, it is found out that the problem of the distribution of the investigated medical equipment is largely solved in the two research lines—as research of the geographic distribution of this medical equipment and as the evaluation of its efficiency. As many studies report, the efficiency of its usage depends not only on their number but also on the types of treatment and diagnostic processes that are set for its use. These processes are influenced by centralised or decentralised systems as well as many regulatory and stabilisation mechanisms in the healthcare system of the countries. Therefore, investigation of the causalities associated with the efficient usage of the medical equipment is very tricky. It is much more difficult to investigate the relationship between the usage of medical equipment and the mortality rate. Mortality is a complex and problematic indicator in terms of healthcare quality assessment. Its condition and development are influenced, among other factors, by the availability of healthcare and the availability of healthcare facilities. Hence, to explicitly quantify the ratio of the individual factors to its state and development is considerably demanding from a methodological point of view too.

## Figures and Tables

**Figure 8:**
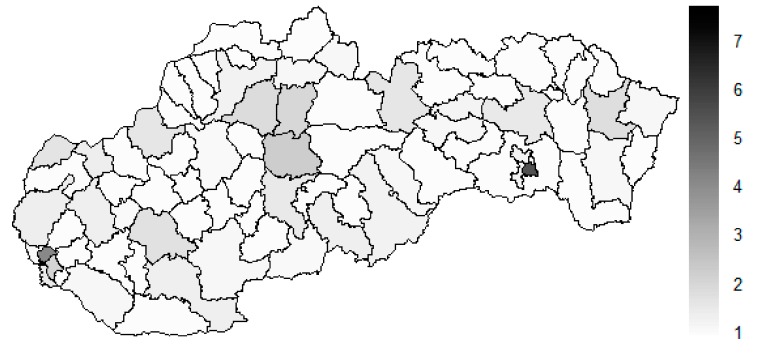
Medical equipment similarity map of the districts in the year 2015. Source: own elaboration by the authors.

**Table 1:** Numbers of individual medical equipment types throughout the whole observed period.

Year	Computed Tomograph	Magnetic Resonance Imaging Scanner
2008	70	33
2009	72	33
2010	78	36
2011	85	37
2012	87	33
2013	86	35
2014	98	44
2015	103	46
2016	100	48
2017	100	51

Source: own elaboration by the authors.

**Table 2:** The ten most extreme districts for the medical equipment distribution.

Rank	2008		2015		Period	
	District	Distance	District	Distance	District	Distance
1.	SK0425	7.66072	SK0101	7.65319	SK0425	7.50630
2.	SK0101	6.60194	SK0425	5.50430	SK0101	6.56821
3.	SK0103	3.46663	SK0103	4.07468	SK0103	4.35243
4.	SK0318	2.36538	SK0321	2.24350	SK0318	2.16281
5.	SK032A	2.01141	SK0102	1.99350	SK0321	1.76306
6.	SK0321	1.97626	SK0318	1.92639	SK0102	1.56271
7.	SK032D	1.93587	SK0316	1.81508	SK032A	1.48217
8.	SK0428	1.50504	SK0412	1.74077	SK0105	1.40086
9.	SK0105	1.50273	SK0233	1.64604	SK0223	1.32614
10.	SK0231	1.46351	SK0216	1.62649	SK0414	1.25496

Source: own elaboration by the authors.

**Table 3:** The ten most extreme districts for the female sex preventable mortality.

Rank	2008		2015		Period	
	District	Distance	District	Distance	District	Distance
1.	SK0214	10.93821	SK0415	12.26389	SK0214	10.97969
2.	SK031B	10.53213	SK0214	11.11897	SK0415	10.92476
3.	SK0226	10.21496	SK0312	10.23134	SK0322	9.99689
4.	SK032B	9.64574	SK0424	10.04509	SK0325	9.15140
5.	SK0327	9.22267	SK0211	9.69639	SK0329	8.86512
6.	SK0231	9.12058	SK0235	9.61492	SK0327	8.77233
7.	SK032C	8.96933	SK0213	9.51470	SK0328	8.61718
8.	SK0429	8.80797	SK0329	8.81856	SK041B	8.30378
9.	SK0424	8.72835	SK0318	8.59940	SK0428	8.21248
10.	SK041C	8.58029	SK0314	8.58039	SK0424	8.20059

Source: own elaboration by the authors.

**Table 4:** The ten most extreme districts for the male sex preventable mortality.

Rank	2008		2015		Period	
	District	Distance	District	Distance	District	Distance
1.	SK031A	12.85978	SK0225	10.38988	SK0413	9.44088
2.	SK0311	10.71538	SK041C	10.37775	SK0322	9.10647
3.	SK0108	10.39499	SK0226	10.23918	SK0429	9.09178
4.	SK0415	10.00868	SK0314	10.04484	SK0103	9.02390
5.	SK0324	9.38335	SK0428	9.11141	SK0421	8.91916
6.	SK0414	9.34402	SK0108	8.95165	SK032A	8.72167
7.	SK0224	9.24618	SK0415	8.94857	SK0415	8.63319
8.	SK0413	8.87369	SK0322	8.92676	SK0311	8.46047
9.	SK0421	8.82257	SK0419	8.92042	SK0105	8.29833
10.	SK041B	8.69976	SK0411	8.83145	SK0221	8.23593

Source: own elaboration by the authors.

**Table 5:** The districts without the explored medical equipment.

Rank	District	Mean Preventable Mortality Rate	Order
1.	SK0225	603.7215	11.
2.	SK0418	638.2817	20.
3.	SK0319	668.8515	28.
4.	SK0221	672.4396	29.
5.	SK0424	678.2058	32.
6.	SK0228	694.1557	35.
7.	SK0108	697.7054	37.
8.	SK0222	701.8454	39.
9.	SK0215	708.0574	40.
10.	SK0107	718.1591	43.
11.	SK0314	719.6071	45.
12.	SK041B	728.0802	47.
13.	SK0324	734.4251	51.
14.	SK032C	763.1765	53.
15.	SK0311	786.6200	58.
16.	SK0415	787.7412	59.
17.	SK0426	794.7751	61.
18.	SK0327	801.2940	63.
19.	SK0237	810.2041	64.
20.	SK0429	816.0116	65.
21.	SK0421	819.4316	67.
22.	SK0322	842.7873	71.
23.	SK0235	850.4742	72.
24.	SK0325	853.4158	73.

Source: own elaboration by the authors.

**Table 6:** The districts with the explored medical equipment.

Rank	District	Mean Preventable Mortality Rate	Order
1.	SK0101	534.9825	1.
2.	SK0229	536.1776	2.
3.	SK0313	560.7561	3.
4.	SK0104	565.4309	4.
5.	SK0411	565.5460	5.
6.	SK0223	572.1936	6.
7.	SK041A	574.5514	7.
8.	SK031A	584.6193	8.
9.	SK0316	591.3131	9.
10.	SK0417	591.4329	10.
11.	SK0315	607.4361	12.
12.	SK0422	610.3983	13.
13.	SK0321	619.7583	14.
14.	SK0224	622.1602	15.
15.	SK0103	622.2497	16.
16.	SK032B	623.5600	17.
17.	SK041C	628.3964	18.
18.	SK0227	630.5185	19.
19.	SK0214	642.0967	21.
20.	SK0416	650.1664	22.
21.	SK0105	655.8287	23.
22.	SK031B	659.2138	24.
23.	SK0102	659.2615	25.
24.	SK0423	664.4445	26.
25.	SK041D	666.1609	27.
26.	SK0217	673.7549	30.
27.	SK032D	677.8043	31.
28.	SK0216	685.0439	33.
29.	SK0233	686.7087	34.
30.	SK0226	695.8348	36.
31.	SK0419	698.4419	38.
32.	SK0414	711.0542	41.
33.	SK0213	717.8904	42.
34.	SK0318	719.4152	44.
35.	SK0106	723.9801	46.
36.	SK0236	729.0889	48.
37.	SK0425	729.9020	49.
38.	SK042A	731.8956	50.
39.	SK0412	755.6380	52.
40.	SK0323	763.8630	54.
41.	SK0317	765.6977	55.
42.	SK0413	779.3107	56.
43.	SK0234	780.1303	57.
44.	SK0427	792.4472	60.
45.	SK0211	797.7173	62.
46.	SK0232	816.3320	66.
47.	SK0212	824.1520	68.
48.	SK0312	836.3790	69.
49.	SK042B	842.5612	70.
50.	SK0326	874.1147	74.
51.	SK0231	884.3118	75.
52.	SK0329	897.5894	76.
53.	SK0428	898.8226	77.
54.	SK032A	909.2262	78.
55.	SK0328	910.1374	79.

Source: own elaboration by the authors.
